# 4-(Methyl­sulfon­yl)piperazin-1-ium chloride

**DOI:** 10.1107/S1600536810001224

**Published:** 2010-01-16

**Authors:** Hoong-Kun Fun, Chin Sing Yeap, C. S. Chidan Kumar, H. S. Yathirajan, B. Narayana

**Affiliations:** a X-ray Crystallography Unit, School of Physics, Universiti Sains Malaysia, 11800 USM, Penang, Malaysia; b Department of Studies in Chemistry, University of Mysore, Manasagangotri, Mysore 570 006, India; c Department of Studies in Chemistry, Mangalore University, Mangalagangotri 574 199, India

## Abstract

In the title mol­ecular salt, C_5_H_13_N_2_O_2_S^+^·Cl^−^, the complete cation is generated by crystallographic mirror symmetry, with both N atoms, the S atom and one C atom lying on the reflecting plane. The chloride ion also lies on the mirror plane. The piperazinium ring adopts a chair conformation and the N—S bond adopts an equatorial orientation. In the crystal structure, the component ions are linked into a three-dimensional framework by inter­molecular N—H⋯Cl and C—H⋯Cl hydrogen bonds.

## Related literature

For medicinal background to piperazine derivatives, see: Dinsmore & Beshore (2002 ▶); Berkheij *et al.* (2005 ▶); Humle & Cherrier (1999 ▶). For related structures, see: Bart *et al.* (1978 ▶); Girisha *et al.* (2008 ▶); Homrighausen & Krause Bauer (2002 ▶); Jin *et al.* (2007 ▶); Kubo *et al.* (2007 ▶); Parkin *et al.* (2004 ▶); Shen *et al.* (2006 ▶), Wang *et al.* (2006 ▶). For ring conformations, see: Cremer & Pople (1975 ▶). For the stability of the temperature controller used for the data collection, see: Cosier & Glazer (1986 ▶).
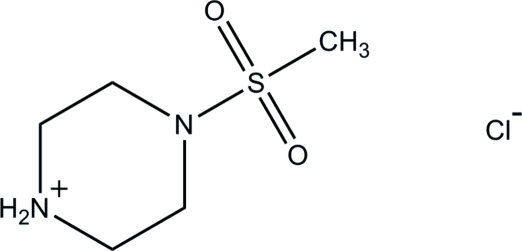


## Experimental

### 

#### Crystal data

C_5_H_13_N_2_O_2_S^+^·Cl^−^
*M* *_r_* = 200.68
Monoclinic, 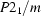

*a* = 6.0231 (1) Å
*b* = 9.1097 (2) Å
*c* = 7.9852 (2) Å
β = 100.700 (1)°
*V* = 430.52 (2) Å^3^
*Z* = 2
Mo *K*α radiation
μ = 0.64 mm^−1^
*T* = 100 K
0.36 × 0.32 × 0.05 mm

#### Data collection

Bruker APEX Duo CCD diffractometer
Absorption correction: multi-scan (*SADABS*; Bruker, 2009 ▶) *T*_min_ = 0.801, *T*_max_ = 0.968
10626 measured reflections
2790 independent reflections
2419 reflections with *I* > 2σ(*I*)
*R*_int_ = 0.022

#### Refinement

*R*[*F* ^2^ > 2σ(*F* ^2^)] = 0.023
*wR*(*F* ^2^) = 0.072
*S* = 1.10
2790 reflections
87 parameters
H atoms treated by a mixture of independent and constrained refinement
Δρ_max_ = 0.49 e Å^−3^
Δρ_min_ = −0.40 e Å^−3^

### 

Data collection: *APEX2* (Bruker, 2009 ▶); cell refinement: *SAINT* (Bruker, 2009 ▶); data reduction: *SAINT* program(s) used to solve structure: *SHELXTL* (Sheldrick, 2008 ▶); program(s) used to refine structure: *SHELXTL*; molecular graphics: *SHELXTL*; software used to prepare material for publication: *SHELXTL* and *PLATON* (Spek, 2009 ▶).

## Supplementary Material

Crystal structure: contains datablocks global, I. DOI: 10.1107/S1600536810001224/hb5306sup1.cif

Structure factors: contains datablocks I. DOI: 10.1107/S1600536810001224/hb5306Isup2.hkl

Additional supplementary materials:  crystallographic information; 3D view; checkCIF report

## Figures and Tables

**Table 1:** Hydrogen-bond geometry (Å, °)

*D*—H⋯*A*	*D*—H	H⋯*A*	*D*⋯*A*	*D*—H⋯*A*
N1—H1*N*1⋯Cl1^i^	0.92 (2)	2.40 (2)	3.1341 (8)	137 (1)
N1—H2*N*1⋯Cl1^ii^	0.93 (2)	2.19 (2)	3.0966 (8)	164 (1)
C1—H1*A*⋯Cl1^iii^	0.953 (12)	2.700 (12)	3.5251 (6)	145.2 (9)
C3—H3*A*⋯Cl1	0.94 (2)	2.65 (2)	3.5487 (10)	160 (2)

Symmetry codes: (i) 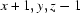; (ii) 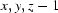; (iii) 
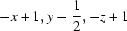
.

**Fig. 1.**
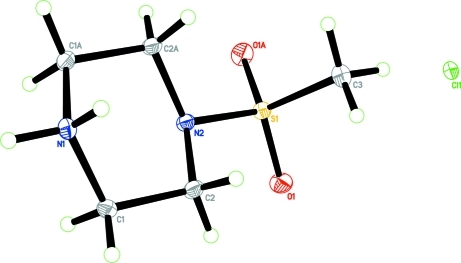
The molecular structure of (I) with 50% probability ellipsoids for the non-H atoms. Atoms with suffix A are generated by the symmetry operation (x, 1/2 - y, z).

**Fig. 2.**
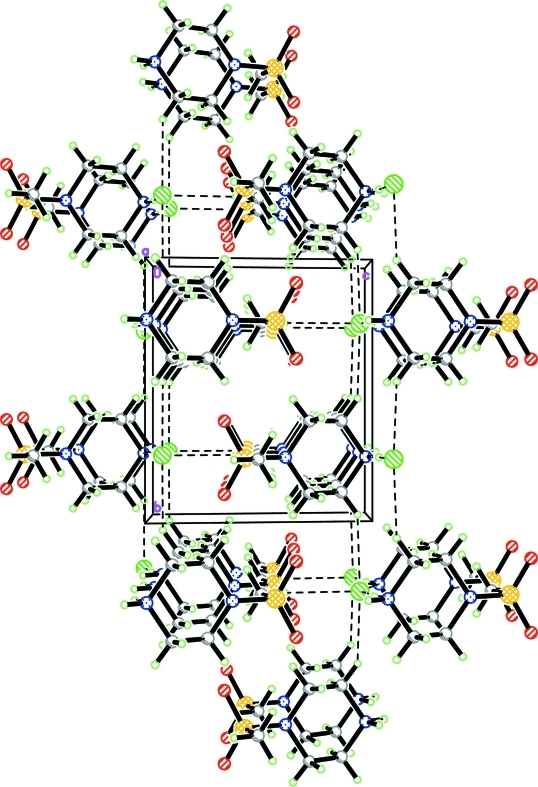
The crystal packing of (I), viewed down the a axis, showing the hydrogen-bonded (dashed lines) three-dimensional framework.

## supplementary crystallographic information

### Comment

Piperazines are among the most important building blocks in today's drug discovery. The piperazine nucleus is capable of binding to multiple receptors with high affinity and therefore piperazine has been classified as a privileged structure (Dinsmore & Beshore, 2002). They are found in biologically active compounds across a number of different therapeutic areas (Berkheij *et al.*, 2005) such as antifungal, antibacterial, antimalarial, antipsychotic, antidepressant and antitumour activity against colon, prostate, breast, lung and leukemia tumors (Humle & Cherrier, 1999). The piperazines are a broad class of chemical compounds, many with important pharmacological properties, which contain a core piperazine functional group. 1-(Methylsulfonyl)piperazine is an important intermediate in synthetic organic chemistry, mainly used as a pharmaceutical intermediate.

The crystal structures of *trans*-2,5-dimethylpiperazine dihydrochloride (Bart *et al.*, 1978), 1-(3-chlorophenyl)-4-(3-chloropropyl)piperazinium chloride (Homrighausen & Krause Bauer, 2002), piperazine (Parkin *et al.*, 2004), 2,2'-(piperazine-1,4-diium-1,4-diyl)diacetate dehydrate (Shen *et al.*, 2006), 1,4-bis(chloroacetyl)piperazine (Wang *et al.*, 2006), 1,4-bis(1-naphthylmethyl) piperazine (Kubo *et al.*, 2007), 1,4-bis(4-chlorobenzo-yl)piperazine (Jin *et al.*, 2007) and 1-benzhydryl-4-(4-chlorophenylsulfonyl) piperazine (Girisha *et al.*, 2008) have been reported. In view of the importance of the title compound, this paper reports its crystal structure.

The asymmetric unit of the title compound contains one-half of a cation and half of a cloride anion (Fig. 1). The Cl1, S1, N1, N2, and C3 atoms are lying on a mirror plane. The piperazinium ring adopts a chair conformation with puckering amplitude Q = 0.5680 (7) Å, θ = 179.90 (7)°, φ = 180 (7)° (Cremer & Pople, 1975). In the crystal structure (Fig. 2), the molecules are linked into a three-dimensional framework by intermolecular hydrogen bonds (Table 1).

### Experimental

The title compound was obtained as a gift sample from *R. L*. Fine Chem., Bangalore, India. The compound was used without further purification. Colourless plates of (I) were obtained from slow evaporation of a methanol solution (m.p.: 489–492 K).

### Refinement

All H atoms were located in a difference Fourier map and refined freely.

### Crystal data

**Table:**

C_5_H_13_N_2_O_2_S^+^·Cl^−^	*F*(000) = 212
*M**_r_* = 200.68	*D*_x_ = 1.548 Mg m^−^^3^
Monoclinic, *P*2_1_/*m*	Mo *K*α radiation, λ = 0.71073 Å
Hall symbol: -P 2yb	Cell parameters from 4890 reflections
*a* = 6.0231 (1) Å	θ = 3.4–40.1°
*b* = 9.1097 (2) Å	µ = 0.64 mm^−^^1^
*c* = 7.9852 (2) Å	*T* = 100 K
β = 100.700 (1)°	Plate, colourless
*V* = 430.52 (2) Å^3^	0.36 × 0.32 × 0.05 mm
*Z* = 2	

### Data collection

**Table:**

Bruker APEX Duo CCD diffractometer	2790 independent reflections
Radiation source: fine-focus sealed tube	2419 reflections with *I* > 2σ(*I*)
graphite	*R*_int_ = 0.022
φ and ω scans	θ_max_ = 40.1°, θ_min_ = 2.6°
Absorption correction: multi-scan (*SADABS*; Bruker, 2009)	*h* = −10→10
*T*_min_ = 0.801, *T*_max_ = 0.968	*k* = −14→16
10626 measured reflections	*l* = −14→14

### Refinement

**Table:**

Refinement on *F*^2^	Primary atom site location: structure-invariant direct methods
Least-squares matrix: full	Secondary atom site location: difference Fourier map
*R*[*F*^2^ > 2σ(*F*^2^)] = 0.023	Hydrogen site location: inferred from neighbouring sites
*wR*(*F*^2^) = 0.072	H atoms treated by a mixture of independent and constrained refinement
*S* = 1.10	*w* = 1/[σ^2^(*F*_o_^2^) + (0.0335*P*)^2^ + 0.0922*P*] where *P* = (*F*_o_^2^ + 2*F*_c_^2^)/3
2790 reflections	(Δ/σ)_max_ = 0.001
87 parameters	Δρ_max_ = 0.49 e Å^−^^3^
0 restraints	Δρ_min_ = −0.40 e Å^−^^3^

### Special details

**Table:**

Experimental. The crystal was placed in the cold stream of an Oxford Cyrosystems Cobra open-flow nitrogen cryostat (Cosier & Glazer, 1986) operating at 100.0 (1) K.
Geometry. All e.s.d.'s (except the e.s.d. in the dihedral angle between two l.s. planes) are estimated using the full covariance matrix. The cell e.s.d.'s are taken into account individually in the estimation of e.s.d.'s in distances, angles and torsion angles; correlations between e.s.d.'s in cell parameters are only used when they are defined by crystal symmetry. An approximate (isotropic) treatment of cell e.s.d.'s is used for estimating e.s.d.'s involving l.s. planes.
Refinement. Refinement of *F*^2^ against ALL reflections. The weighted *R*-factor *wR* and goodness of fit *S* are based on *F*^2^, conventional *R*-factors *R* are based on *F*, with *F* set to zero for negative *F*^2^. The threshold expression of *F*^2^ > σ(*F*^2^) is used only for calculating *R*-factors(gt) *etc*. and is not relevant to the choice of reflections for refinement. *R*-factors based on *F*^2^ are statistically about twice as large as those based on *F*, and *R*- factors based on ALL data will be even larger.

### Fractional atomic coordinates and isotropic or equivalent isotropic displacement parameters (Å^2^)

**Table:**

	*x*	*y*	*z*	*U*_iso_*/*U*_eq_	
Cl1	0.30674 (3)	0.2500	0.93448 (3)	0.01195 (5)	
S1	0.69091 (3)	0.2500	0.56598 (2)	0.00951 (5)	
N1	0.82854 (12)	0.2500	0.03611 (9)	0.01007 (11)	
N2	0.79320 (12)	0.2500	0.38830 (9)	0.00974 (11)	
O1	0.75935 (9)	0.11396 (6)	0.65235 (6)	0.01498 (9)	
C1	0.87784 (10)	0.11522 (7)	0.14202 (8)	0.01165 (9)	
C2	0.74465 (10)	0.11465 (7)	0.28537 (8)	0.01168 (9)	
C3	0.39411 (15)	0.2500	0.50705 (12)	0.01332 (13)	
H1A	0.8351 (18)	0.0315 (13)	0.0719 (14)	0.012 (2)*	
H1B	1.040 (2)	0.1186 (13)	0.1845 (16)	0.016 (3)*	
H2A	0.583 (2)	0.1017 (14)	0.2371 (15)	0.018 (3)*	
H2B	0.789 (2)	0.0335 (16)	0.3554 (17)	0.027 (3)*	
H3A	0.331 (3)	0.2500	0.606 (2)	0.019 (4)*	
H3B	0.350 (2)	0.3381 (15)	0.4460 (16)	0.025 (3)*	
H1N1	0.914 (3)	0.2500	−0.048 (2)	0.019 (4)*	
H2N1	0.677 (3)	0.2500	−0.017 (2)	0.021 (4)*	

### Atomic displacement parameters (Å^2^)

**Table:**

	*U*^11^	*U*^22^	*U*^33^	*U*^12^	*U*^13^	*U*^23^
Cl1	0.00886 (7)	0.01261 (8)	0.01485 (9)	0.000	0.00346 (6)	0.000
S1	0.01110 (8)	0.01033 (8)	0.00696 (8)	0.000	0.00128 (6)	0.000
N1	0.0098 (2)	0.0115 (3)	0.0095 (3)	0.000	0.00316 (19)	0.000
N2	0.0126 (2)	0.0084 (2)	0.0087 (2)	0.000	0.0032 (2)	0.000
O1	0.01874 (19)	0.01499 (19)	0.01109 (18)	0.00356 (16)	0.00245 (15)	0.00480 (15)
C1	0.0148 (2)	0.00900 (19)	0.0122 (2)	0.00097 (17)	0.00547 (17)	−0.00041 (17)
C2	0.0162 (2)	0.0083 (2)	0.0117 (2)	−0.00109 (16)	0.00571 (17)	−0.00060 (16)
C3	0.0115 (3)	0.0166 (3)	0.0121 (3)	0.000	0.0027 (2)	0.000

### Geometric parameters (Å, °)

**Table:**

S1—O1^i^	1.4408 (5)	N2—C2^i^	1.4806 (7)
S1—O1	1.4408 (5)	C1—C2	1.5148 (8)
S1—N2	1.6484 (7)	C1—H1A	0.953 (11)
S1—C3	1.7621 (9)	C1—H1B	0.976 (12)
N1—C1	1.4892 (7)	C2—H2A	0.983 (13)
N1—C1^i^	1.4892 (7)	C2—H2B	0.935 (14)
N1—H1N1	0.920 (17)	C3—H3A	0.941 (18)
N1—H2N1	0.933 (19)	C3—H3B	0.951 (14)
N2—C2	1.4806 (7)		
			
O1^i^—S1—O1	118.67 (4)	N1—C1—C2	110.75 (5)
O1^i^—S1—N2	107.01 (2)	N1—C1—H1A	108.8 (7)
O1—S1—N2	107.01 (2)	C2—C1—H1A	108.5 (6)
O1^i^—S1—C3	108.28 (3)	N1—C1—H1B	104.6 (7)
O1—S1—C3	108.29 (3)	C2—C1—H1B	112.1 (7)
N2—S1—C3	107.03 (4)	H1A—C1—H1B	112.0 (9)
C1—N1—C1^i^	111.07 (7)	N2—C2—C1	109.81 (5)
C1—N1—H1N1	109.7 (5)	N2—C2—H2A	113.4 (7)
C1^i^—N1—H1N1	109.7 (5)	C1—C2—H2A	109.1 (7)
C1—N1—H2N1	109.5 (5)	N2—C2—H2B	108.7 (8)
C1^i^—N1—H2N1	109.5 (5)	C1—C2—H2B	108.7 (7)
H1N1—N1—H2N1	107.3 (15)	H2A—C2—H2B	107.1 (10)
C2—N2—C2^i^	112.77 (7)	S1—C3—H3A	108.9 (11)
C2—N2—S1	114.27 (4)	S1—C3—H3B	108.1 (8)
C2^i^—N2—S1	114.27 (4)	H3A—C3—H3B	108.3 (9)
			
O1^i^—S1—N2—C2	178.10 (5)	C3—S1—N2—C2^i^	66.00 (5)
O1—S1—N2—C2	49.91 (6)	C1^i^—N1—C1—C2	56.95 (8)
C3—S1—N2—C2	−65.99 (5)	C2^i^—N2—C2—C1	56.73 (8)
O1^i^—S1—N2—C2^i^	−49.91 (6)	S1—N2—C2—C1	−170.56 (4)
O1—S1—N2—C2^i^	−178.10 (5)	N1—C1—C2—N2	−55.89 (7)

Symmetry codes: (i) *x*, −*y*+1/2, *z*.

### Hydrogen-bond geometry (Å, °)

**Table:**

*D*—H···*A*	*D*—H	H···*A*	*D*···*A*	*D*—H···*A*
N1—H1N1···Cl1^ii^	0.92 (2)	2.40 (2)	3.1341 (8)	137 (1)
N1—H2N1···Cl1^iii^	0.93 (2)	2.19 (2)	3.0966 (8)	164 (1)
C1—H1A···Cl1^iv^	0.953 (12)	2.700 (12)	3.5251 (6)	145.2 (9)
C3—H3A···Cl1	0.94 (2)	2.65 (2)	3.5487 (10)	160 (2)

Symmetry codes: (ii) *x*+1, *y*, *z*−1; (iii) *x*, *y*, *z*−1; (iv) −*x*+1, *y*−1/2, −*z*+1.
